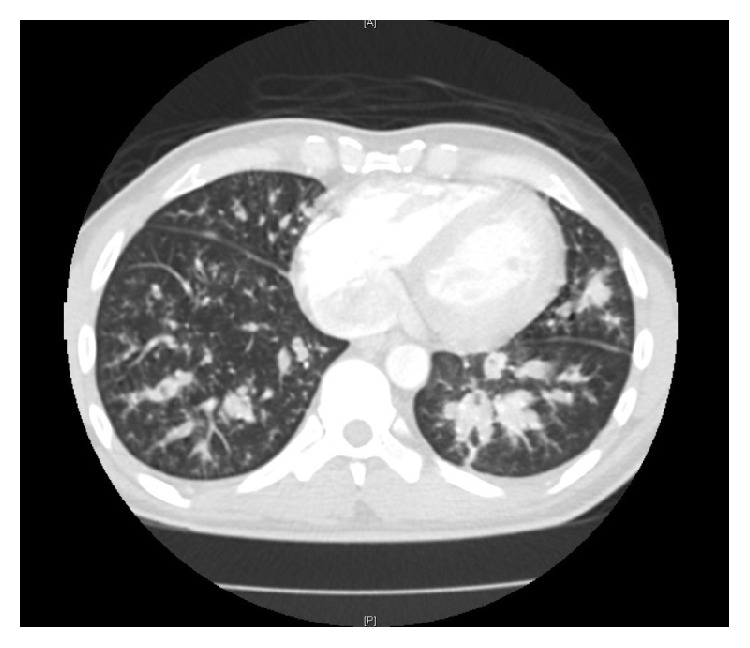# Humoral Immunity in Bronchiectasis: Finding Good's Syndrome

**DOI:** 10.1155/2015/531731

**Published:** 2015-12-29

**Authors:** C. Pu, S. Sukhal, S. Fakhran

**Affiliations:** ^1^Department of Medicine, John H. Stroger Jr. Hospital of Cook County, Chicago, IL 60612, USA; ^2^Division of Pulmonary, Critical Care and Sleep Medicine, John H. Stroger Jr. Hospital of Cook County, Chicago, IL 60612, USA

## Abstract

We present a case of a 37-year-old man with a past history of a surgically removed thymoma, who presented with recurrent pulmonary infections and bronchiectasis. On further testing, he was found to have low total immunoglobulin levels, a constellation of findings known as Good's syndrome. He responded well to immunoglobulin replacement, in addition to the usual treatments for bronchiectasis. We present this case to emphasize the association of bronchiectasis, low immunoglobulins, and thymomas and the role of immunoglobulin replacement as a treatment option.

## 1. Introduction

Good syndrome is a rare disease that comprises thymoma and humoral immunodeficiency. It tends to manifest in middle age leading to significant morbidity and mortality.

## 2. Case

**Figure 1:**
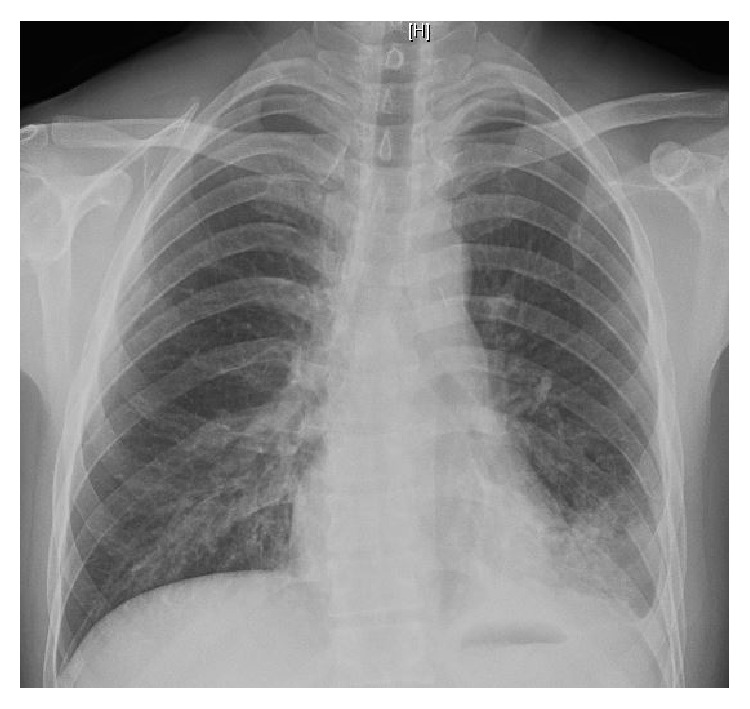


A 37-year-old man was referred to the pulmonary clinic for recurrent episodes of cough with purulent expectoration, low grade fevers, and lethargy. He was treated with short courses of antibiotics over the last 2 months. He denied dyspnea, wheezing, or chest pain. His past medical history was significant for type AB thymoma diagnosed two years ago which was treated with thymectomy and adjuvant radiotherapy. He worked as a gardener, did not smoke, and had no prior inhalational occupational exposure. He had a healthy childhood and had no significant medical problems until he was diagnosed with thymoma. He was born in Mexico but lived in Chicago for the last twenty years. Physical examination was notable for left lung base crackles and finger clubbing. The rest of his physical examination was unremarkable. His white cell count was 12000 cells/*μ*L with 90% neutrophils. Multiple prior sputum bacterial cultures were negative. Chest radiography (see Figure 1) revealed a left lower lobe infiltrate while a contrast enhanced computed tomography of the chest (Figure 2) showed bilateral lower lobe bronchiectasis with endobronchial mucus plugging. He was diagnosed with bronchiectasis and was treated with antibiotics, inhaled bronchodilators, and airway clearance therapies. Over the next few months, he had variable success with treatment requiring multiple courses of antibiotics for exacerbations. Further workup for bronchiectasis found low total immunoglobulin (Ig) IgG 140 mg/dL (normal 694–378 mg/dL), IgA 7 mg/dL (68–378 mg/dL), and IgM 8 mg/dL (77–220 mg/dL). Total IgE was less than 2 mg/dL and* Aspergillus fumigatus* IgE levels were undetectable. Analytic cytometry analysis detected decrease in CD19/20+ B-cells. T-cells present showed coexpression of all appropriate antigens tested. Alpha-1 antitrypsin level was normal; anti-neutrophilic antibody and rheumatoid factor were negative. Bronchoalveolar lavage of the left lower lobe was inflammatory with high neutrophils but bacterial, mycobacterial, and fungal smears and cultures were negative.

He was diagnosed with Good's syndrome as he had hypogammaglobulinemia in the context of a thymoma with recurrent pulmonary infections leading to bronchiectasis. He was started on immunoglobulin replacement therapy with monthly IVIG (intravenous immunoglobulin) infusions. His IgG level improved to 540 mg/dL. Since starting IVIG treatment, he has not had any exacerbations of bronchiectasis and has been doing well.

## 3. Discussion

While 53% of bronchiectasis in adults is idiopathic, 7% of patients with bronchiectasis have humoral immune defects [1]. The most common immune deficiency diseases causing recurrent pulmonary infections and bronchiectasis are common variable immune deficiency (CVID) and X-linked agammaglobulinemia (XLA). Bronchiectasis is attributable to CVID in 0.7–2.4% of adults and 2–10% of children [2]. X-linked agammaglobulinemia is very rare in adults but accounts for 3% of childhood bronchiectasis [2]. The British Thoracic Society guidelines for approach to patients with non-Cystic Fibrosis bronchiectasis recommends that all patients with bronchiectasis be screened for immunodeficiency. The first-line screening tests include serum IgG, IgA, IgM, and serum electrophoresis [3]. If antibody levels are normal but clinical suspicion remains high, humoral response against tetanus toxoid,* Streptococcus pneumoniae,* and* Haemophilus influenzae* capsular polysaccharide [4–6] should be tested by antibody assays after immunization.

The association of thymoma with adult onset hypogammaglobulinemia was first described by Dr. Good in 1954 [7]. It is a rare entity, with 281 cases described in literature. The incidence of thymoma is 0.15 cases per 100,0000 in the United States [8] and about 6–11% of patients with a thymoma have hypogammaglobulinemia [8, 9]. Good's syndrome (GS) usually manifests in middle age and the mean age of diagnosis is 59 years. The recognition of a thymoma predates immune deficiency in almost 42% of patients [10]. There are no clear diagnostic criteria for GS, but it is a distinct entity described by World Health Organization/International Union of Immunological Societies as a primary immunodeficiency with thymoma and hypogammaglobulinemia similar to CVID [11]. The exact pathogenesis of immunodeficiency in GS is unclear but there are two major hypotheses. The first postulates that cytokines produced by bone marrow stromal cells influence both thymic and B-cell precursor growth and differentiation [12]. This is based on murine studies showing that limitin, an interferon-like cytokine produced by bone marrow stromal cell line, preferentially inhibits precursor B-cell growth and differentiation [13]. The second hypothesis is that thymic T-cells directly inhibit B-cell immunoglobulin production [14]. This theory is derived from studies of paraneoplastic phenomena in thymomas, where T-cells or autoantibodies directly or indirectly inhibit erythropoiesis [15]. Genetic studies show a possible role of Transmembrane Activator and CAML interactor (TACI) mutation in B-cells and plasma cells in pathogenesis of both CVID and GS [16, 17]. Supporting the role of autoantibodies in its pathogenesis, Good's syndrome also has many autoimmune manifestations, such as pure red cell aplasia (34.8%), aplastic anemia (7.9), macrocytic anemia (5.6%), and autoimmune hemolytic anemia (3.4) [10]. However, myasthenia gravis is less common in GS (15.7%) than in thymoma alone (25–40%) [10, 18–20].

Available data suggests that the prognosis of GS is worse than other immunodeficiencies, with 70% of patients with GS being alive after 5 years, while only 33% are alive after 10 years [10]. Furthermore, bronchiectasis caused by thymoma associated hypogammaglobulinemia has a higher mortality rate than other primary humoral deficiencies [21]. Although there are no formal studies of immunoglobulin replacement in patients with Good's syndrome, it is a recommended treatment modality [14]. IVIG replacement has been shown to reduce the incidence of pulmonary infections and progression of lung injury in other hypogammaglobinemic states such as XLA and CVID [22–24]. IVIG replacement reduces the rate of bacterial lung infection in XLA from 1.67 episodes to 0.45 episodes per patient per year and in CVID from 1.11 to 0.58 episodes per patient per year [25]. Orange et al. found that, in patients with primary immunodeficiency on monthly IVIG infusion, keeping a higher IgG trough level lowers the risk of pneumonia [26]. After three months of follow-up after initiation of IVIG replacement and standard bronchiectasis treatment, our patient has been stable without recurrent infections.

## Figures and Tables

**Figure 2:**